# Do Ceramic Femoral Heads Reduce Taper Fretting Corrosion in Hip Arthroplasty? A Retrieval Study

**DOI:** 10.1007/s11999-013-3096-2

**Published:** 2013-06-13

**Authors:** Steven M. Kurtz, Sevi B. Kocagöz, Josa A. Hanzlik, Richard J. Underwood, Jeremy L. Gilbert, Daniel W. MacDonald, Gwo-Chin Lee, Michael A. Mont, Matthew J. Kraay, Gregg R. Klein, Javad Parvizi, Clare M. Rimnac

**Affiliations:** 1 Implant Research Center, School of Biomedical Engineering, Science, and Heath Systems, Drexel University, 3401 Market Street, Suite 345, Philadelphia, PA 19104 USA; 2 Exponent, Inc, Philadelphia, PA USA; 3 Syracuse Biomaterials Institute, Syracuse University, Syracuse, NY USA; 4 Penn Orthopaedics, Presbyterian Medical Center of Philadelphia, Philadelphia, PA USA; 5 Rubin Institute for Advanced Orthopedics, Sinai Hospital of Baltimore, Baltimore, MD USA; 6 University Hospitals Case Medical Center, Cleveland, OH USA; 7 Hartzband Center for Hip and Knee Replacement, Hackensack, NJ USA; 8 Rothman Institute, Thomas Jefferson University Hospital, Philadelphia, PA USA; 9 Mechanical and Aerospace Engineering, Case Western Reserve University, Cleveland, OH USA

## Abstract

**Background:**

Previous studies regarding modular head-neck taper corrosion were largely based on cobalt chrome (CoCr) alloy femoral heads. Less is known about head-neck taper corrosion with ceramic femoral heads.

**Questions/purposes:**

We asked (1) whether ceramic heads resulted in less taper corrosion than CoCr heads; (2) what device and patient factors influence taper fretting corrosion; and (3) whether the mechanism of taper fretting corrosion in ceramic heads differs from that in CoCr heads.

**Methods:**

One hundred femoral head-stem pairs were analyzed for evidence of fretting and corrosion using a visual scoring technique based on the severity and extent of fretting and corrosion damage observed at the taper. A matched cohort design was used in which 50 ceramic head-stem pairs were matched with 50 CoCr head-stem pairs based on implantation time, lateral offset, stem design, and flexural rigidity.

**Results:**

Fretting and corrosion scores were lower for the stems in the ceramic head cohort (p = 0.03). Stem alloy (p = 0.004) and lower stem flexural rigidity (Spearman’s rho = −0.32, p = 0.02) predicted stem fretting and corrosion damage in the ceramic head cohort but not in the metal head cohort. The mechanism of mechanically assisted crevice corrosion was similar in both cohorts although in the case of ceramic femoral heads, only one of the two surfaces (the male metal taper) engaged in the oxide abrasion and repassivation process.

**Conclusions:**

The results suggest that by using a ceramic femoral head, CoCr fretting and corrosion from the modular head-neck taper may be mitigated but not eliminated.

**Clinical Relevance:**

The findings of this study support further study of the role of ceramic heads in potentially reducing femoral taper corrosion.

## Introduction

Taper corrosion in THA was identified as a clinical concern in the 1980s to 1990s [2, 5, 7, 14, 15, 24, 25] and was believed to have been addressed. However, implant corrosion has recently been reintroduced as a clinical issue [3, 8, 16–18]. There is consensus that the mechanism of taper corrosion is best characterized as mechanically assisted crevice corrosion [7, 14, 22]. Although fundamentally a crevice corrosion problem, mechanical fretting and wear also contribute by disrupting the atomically thin, protective oxide layers that border the crevice environment [7, 9, 14]. When the underlying metallic substrate is exposed by mechanical damage to the in vivo environment, rapid repassivation of the metal surfaces alters its voltage and acidifies the solution trapped in the taper crevice. Thus, the electrochemistry of the head and stem alloys as well as the solution chemistry of the taper crevice are determinants of taper corrosion [7, 14]. For the modular head-neck connection, the crevice is the space between two opposing taper surfaces (ie, where no asperity-asperity contact is present and solution can reside). This is effectively a crack-like fluid-filled environment in electrochemical contact with the outside solution, wherein large changes in solution chemistry and crevice-type corrosion reactions can occur.

Taper corrosion depends on the dimensions and shape of the crevice at the taper interface and the complex interplay of metallurgical, chemical, electrical, and tribological factors [7, 14, 22]. A previous, multicenter retrieval analysis of head-neck taper corrosion by Goldberg et al. [9] documented that the combination of dissimilar alloys, metallurgical condition of the alloys, implantation time, and flexural rigidity of the femoral neck were predictors of corrosion of the neck and head. Implantation time and dissimilar alloys were also identified as important variables in a related retrieval study [5]. More recently, a multicenter retrieval study of modular taper connections in contemporary metal-on-metal (MOM) bearings also found that implantation time, lateral offset, femoral stem modularity, and dissimilar alloys were predictors of taper corrosion [11]. Evaluation of corrosion in the past, and for the current study, is conducted using a visual scoring method with a scale of 1 through 4 where 1 is least severe and 4 is most severe.

To date, the body of knowledge regarding taper corrosion, including the majority of previous retrieval studies of retrieved tapers, is based on cobalt chrome (CoCr) alloy femoral heads on a metallic stem in either metal-on-polyethylene (M-PE) or MOM bearings. Less is known about taper corrosion with ceramic heads on a metallic stem in ceramic-on-polyethylene (C-PE) or ceramic-on-ceramic (COC) bearings or how stem taper corrosion differs between ceramic and CoCr heads [10, 13, 25]. Previous studies are limited to a case study [25], studies with significant confounding factors (ie, additional modular junctions) [13], or limited to components and designs that are no longer commercially relevant [10].

In the current study we asked (1) whether ceramic heads resulted in less taper corrosion than CoCr heads; (2) what device and patient factors exert a significant influence on taper fretting corrosion; and (3) whether the mechanism of taper fretting corrosion in ceramic heads differs from that in CoCr heads.

## Materials and Methods

### Study Design, Cohort Selection, and Clinical Information

We based our matched cohort design on the combined retrieval collections of two academic engineering-based programs working in collaboration with 12 clinical revision centers from the northeast, midwest, south, and western regions of the United States. Retrievals were collected as part of a 12-year ongoing institutional review board-approved revision and retrieval program. An a priori power analysis was conducted and revealed that a sample size of 100 was more than sufficient to detect a difference in corrosion score of 1 between the metal and ceramic cohorts (power = 99.9%). Thus, a total sample size of 100 retrieval cases was judged to be adequate based on this analysis and previous research involving taper corrosion in MOM retrievals [11], in which researchers detected significant differences in taper corrosion between study groups using a sample size of approximately 100 retrievals.

From our combined interinstitutional database of over 2000 THAs, we first identified 96 sets of matched ceramic head/femoral stem taper pairs. The identified sets were restricted to ceramic heads that were produced by the same supplier (Ceramtec GmbH, Plochingen, Germany) and distributed by major manufacturers in North America. According to the supplier, the geometric specifications of the femoral taper angle as well as surface roughness that mates with the bore of the ceramic head have remained unchanged since the 1980s. However, the ceramic material has evolved over time such that the 96 sets included two grades of alumina (Biolox and Biolox^®^ forte; Ceramtec GmbH, Plochingen, Germany) and zirconia-toughened alumina (Biolox^®^ delta) ceramic heads. The transition from Biolox^®^ to Biolox^®^ forte grades of alumina took place in 1995 and Biolox^®^ delta was clinically introduced in 2003 in Europe and 2005 in the United States. From this set, we selected the ceramic-metal taper cohort. Because previous studies have shown implantation time to be one of the most important variables related to taper corrosion [5, 7, 9], we selected the ceramic-metal taper cohort to consist of the 50 sets (Biolox^®^ [n = 5], Biolox^®^ forte [n = 30], and Biolox^®^ delta [n = 15]) with the longest implantation time and which could be matched with a metal-metal taper cohort (described subsequently). The ceramic-metal taper cohort included both COC and/or C-PE bearings (Table 1). The majority of the components for this study were uncemented (94 of 100) with the cemented components having COC (n = 1), C-PE (n = 2), and M-PE (n = 3) bearing couples. Given that cement was present in three samples for both study groups, cement is not considered to be a confounding factor for this study. We excluded 11 prostheses with a modular femoral stem from the study because of previous research suggesting that modular tapers were associated with increased femoral head corrosion in MOM bearings [11].

**Table 1:** Patient and device information corresponding to the ceramic-metal taper cohort

Case number	Implantation time (years)	Age at insertion (years)	Sex	BMI (kg/m^2^)	Maximum UCLA score	Revision reason	Head size (mm)	Lateral offset (mm)	Manufacturer	Stem type	Stem alloy	Stem taper angle	Flexural rigidity 10^9^·Nm^2^
1	0.5	75	M	27	7	Loosening	36	41.5	Stryker	Accolade	TMZF	V40	89
2	0.5	31	M	33	5	Infection	36	39	Stryker	Accolade	TMZF	V40	89
3	0.5	48	M	42	2	Loosening	36	50.3	Stryker	Accolade	TMZF	V40	89
4	0.6	N/A	M	N/A	N/A	Loosening	32	44	Stryker	Accolade	TMZF	V40	89
5	0.6	39	M	41	N/A	Infection	36	37	Stryker	Accolade	TMZF	V40	89
6	0.6	46	F	19	4	Infection	36	49	DePuy	Corail	Ti-6Al-4V	12/14	185
7	0.7	49	M	27	2	Loosening	32	40	Zimmer	M/L Taper	Ti-6Al-4V	12/14	202
8	0.8	52	M	38	6	Loosening	32	41	Zimmer	M/L Taper	Ti-6Al-4V	12/14	202
9	0.8	49	M	35	5	Loosening	32	48	Stryker	Accolade	TMZF	V40	89
10	0.9	49	M	N/A	N/A	Loosening	32	44.2	Stryker	Accolade	TMZF	V40	89
11	1.0	53	M	27	4	Infection	36	39	Stryker	Omnifit	Ti-6Al-4V	V40	185
12	1.0	53	M	28	7	Loosening	36	33.4	Stryker	Accolade	TMZF	V40	89
13	1.0	55	M	N/A	5	Loosening	36	39	Stryker	Accolade	TMZF	V40	89
14	1.1	43	M	21	8	Infection	36	58	DePuy	Corail	Ti-6Al-4V	12/14	185
15	1.2	68	F	39	4	Instability	32	38	Zimmer	M/L Taper	Ti-6Al-4V	12/14	202
16	1.2	50	M	34	6	Pain	32	38	DePuy	Tri-Lock	Ti-6Al-4V	12/14	185
17	1.2	55	F	31	8	Loosening	28	44	Stryker	Accolade	TMZF	V40	89
18	1.4	41	M	44	6	Loosening	32	34	Stryker	Accolade	TMZF	V40	89
19	1.5	57	M	30	3	Infection	32	42	Smith & Nephew	Anthology	Ti-6Al-4V	12/14	185
20	1.6	48	M	29	4	Infection	36	39	Stryker	Accolade	TMZF	V40	89
21	1.7	N/A	M	N/A	6	Loosening	32	49	Stryker	Accolade	TMZF	V40	89
22	1.7	50	F	N/A	N/A	Loosening	32	32.1	Stryker	Accolade	TMZF	V40	89
23	1.9	53	F	27	N/A	Malalignment	28	40	Stryker	Accolade	TMZF	V40	89
24	1.9	63	M	28	4	Loosening	36	49	Stryker	Accolade	TMZF	V40	89
25	2.0	53	M	N/A	N/A	Loosening	32	38	Stryker	Accolade	TMZF	V40	89
26	2.0	61	M	28	6	Loosening	36	48	Stryker	Accolade	TMZF	V40	89
27	2.1	50	F	26	7	Loosening	32	32	Stryker	Accolade	TMZF	V40	89
28	2.3	32	M	34	10	Loosening	32	49	Stryker	Accolade	TMZF	V40	89
29	2.3	72	F	22	N/A	Malposition	32	44	Zimmer	Versys FMT	Ti-6Al-4V	12/14	202
30	2.3	34	M	34	3	Infection	32	45	Smith & Nephew	Echelon	CoCr	12/14	358
31	2.4	71	M	N/A	N/A	Loosening	32	38	Stryker	Accolade	TMZF	V40	89
32	2.5	51	M	29	2	Infection	32	40	Smith & Nephew	Echelon	CoCr	12/14	353
33	2.5	57	F	21	6	Loosening	28	36	Encore	Linear	Ti-6Al-4V	12/14	220
34	2.5	49	M	24	6	Infection	32	27	DePuy	Tri-Lock	Ti-6Al-4V	12/14	185
35	2.9	N/A	F	25	5	Loosening	32	41	Zimmer	Versys FMT	Ti-6Al-4V	12/14	190
36	3.4	59	M	28	6	Loosening	36	52	Stryker	Accolade	TMZF	V40	89
37	3.5	48	F	32	7	Loosening	32	50	Stryker	Accolade	TMZF	V40	89
38	3.6	76	F	22	8	Periprosthetic fracture	28	32	Stryker	Accolade	TMZF	V40	89
39	4.5	56	M	52	N/A	Loosening	36	38	Stryker	Accolade	TMZF	V40	89
40	5.1	42	F	26	6	Malalignment	28	44	Wright Medical	Perfecta	Ti-6Al-4V	12/14	252
41	5.4	66	M	28	7	Loosening	28	34	Stryker	Accolade	TMZF	V40	89
42	5.6	46	M	18	2	Pain	36	59	DePuy	Corail	Ti-6Al-4V	12/14	179
43	5.9	58	M	37	7	Loosening	32	49	Stryker	Accolade	TMZF	V40	89
44	6.3	43	M	27	10	Osteolysis	32	35	Smith & Nephew, Richards	Spectron EF	CoCr	12/14	353
45	6.3	47	F	32	8	Infection	32	39	Stryker	Accolade	TMZF	V40	89
46	7.6	47	F	21	N/A	Infection	32	45	DePuy	Solution	CoCr	12/14	676
47	8.8	47	F	21	N/A	Infection	32	46	DePuy	Solution	CoCr	12/14	676
48	14.3	52	F	38	N/A	Femoral loosening	32	36	Wright Medical, Dow Corning Wright	Infinity	Ti-6Al-4V	12/14	226
49	14.6	64	F	34	N/A	Loosening	32	46	Zimmer	Muller modified	CoCr	12/14	385
50	17.8	48	M	33	8	PE wear	32	41	Smith & Nephew, Richards	Opti-Fix	Ti-6Al-4V	14/16	328

BMI = body mass index; N/A = not available; M = male; F = female; PE = polyethylene; CoCr = cobalt-chromium.

We identified the matched cohort of 50 metal-metal tapered head-stem components from M-PE bearings (Table 2). The metal femoral head in the metal-metal taper cohort was always composed of CoCr. The metallic head and stem material compositions of all samples were confirmed using an x-ray fluorescence detector (Niton XL3t GOLDD+; Thermo Scientific, Waltham, MA, USA). Devices in the ceramic-metal taper cohort were matched to create the metal-metal taper cohort based on the following three criteria (in order of importance) based on significant variables published in previous retrieval studies of taper corrosion [9, 11]: (1) implantation time (most important); (2) stem neck flexural rigidity; and (3) lateral offset (least important). Although not specifically matched for, the resulting cohorts had similar head diameters (median = 32 mm and mean = 33 mm for both cohorts). In this study, the CoCr heads had the same manufacturer as the stems they were implanted with, eliminating manufacturer mixing as a confounding factor.

**Table 2:** Patient and device information corresponding to the matched metal-metal taper cohort

Case number	Implantation time (years)	Age at insertion (years)	Sex	BMI (kg/m^2^)	Maximum UCLA score	Revision reason	Head size (mm)	Lateral offset (mm)	Manufacturer	Stem type	Stem alloy	Trunnion type	Flexural rigidity 10^9^·Nm^2^
1	0.9	51	F	23	6	Infection	28	38	Stryker	Accolade	TMZF	V40	89
2	0.5	70	F	32	3	Loosening	32	38	Stryker	Accolade	TMZF	V40	89
3	0.6	79	F	N/A	N/A	Loosening	32	46	Stryker	Accolade	TMZF	V40	89
4	0.6	64	M	31	7	Loosening	40	44	Stryker	Accolade	TMZF	V40	89
5	0.7	46	M	37	8	Loosening	32	39	Stryker	Accolade	TMZF	V40	89
6	1.0	N/A	F	35	N/A	Infection	32	44	Zimmer	Versys FMT	Ti-6Al-4V	12/14	202
7	0.5	59	M	N/A	8	Infection	36	44	Zimmer	M/L Taper	Ti-6Al-4V	12/14	202
8	0.8	52	F	28	6	Infection	36	48	Zimmer	M/L Taper	Ti-6Al-4V	12/14	202
9	1.4	42	M	27	5	Infection	32	58	Stryker	Accolade	TMZF	V40	247
10	1.0	61	M	29	5	Loosening	36	55	Stryker	Accolade	TMZF	V40	89
11	1.1	65	F	33	4	Infection	36	39	Zimmer	Versys FMT	Ti-6Al-4V	12/14	202
12	1.1	59	F	22	N/A	Infection	36	32	Stryker	Accolade	TMZF	V40	89
13	0.8	67	M	38	4	Infection	36	33	Stryker	Accolade	TMZF	V40	89
14	1.2	47	F	40	2	Infection	36	65	DePuy	Corail	Ti-6Al-4V	12/14	196
15	1.0	65	M	26	5	Loosening	32	51	Zimmer	M/L Taper	Ti-6Al-4V	12/14	202
16	1.3	67	F	25	2	Loosening	32	40	DePuy	Tri-Lock	Ti-6Al-4V	12/14	185
17	1.0	56	M	30	8	Loosening	40	45	Stryker	Accolade	TMZF	V40	89
18	1.4	46	F	N/A	2	Loosening	28	34	Stryker	Accolade	TMZF	V40	89
19	1.5	83	M	23	8	Infection	32	49	Zimmer	M/L Taper	Ti-6Al-4V	12/14	202
20	0.8	53	F	N/A	7	Loosening	32	41	Stryker	Accolade	TMZF	V40	89
21	1.8	55	M	36	4	Loosening	36	46	Stryker	Accolade	TMZF	V40	89
22	1.8	82	M	27	3	Loosening	32	33	Stryker	Accolade	TMZF	V40	89
23	1.7	57	F	29	4	Infection	40	43	Stryker	Accolade	TMZF	V40	89
24	1.9	57	M	28	N/A	Loosening	32	48	Stryker	Accolade	TMZF	V40	89
25	1.0	76	F	31	4	Loosening	32	44	Stryker	Accolade	TMZF	V40	89
26	2.2	N/A	F	22	4	Loosening	28	47	Stryker	Accolade	TMZF	V40	236
27	2.0	59	F	43	6	Loosening	36	34	Stryker	Accolade	TMZF	V40	89
28	1.9	45	M	45	7	Loosening	32	44	Stryker	Accolade	TMZF	V40	89
29	2.0	63	M	35	6	Infection	32	49	Zimmer	Versys FMT	Ti-6Al-4V	12/14	202
30	2.5	N/A	M	23	2	Infection	40	42	Zimmer	Versys Beaded Fullcoat	CoCr	12/14	385
31	1.1	57	F	23	N/A	Infection	36	35	Stryker	Accolade	TMZF	V40	89
32	2.5	76	M	22	N/A	Infection	28	46	Zimmer	Harris Precoat	CoCr	6°	180
33	2.3	49	F	35	6	Infection	40	65	DePuy	Corail	Ti-6Al-4V	12/14	202
34	2.2	61	F	23	7	N/A	28	42	DePuy	Tri-Lock	Ti-6Al-4V	12/14	185
35	2.3	63	F	51	4	Infection	32	39	Zimmer	Versys FMT	Ti-6Al-4V	12/14	202
36	2.7	55	M	29	6	Loosening	32	50	Stryker	Accolade	TMZF	V40	89
37	2.3	74	M	34	9	Loosening	32	48	Stryker	Accolade	TMZF	V40	89
38	3.6	50	M	20		Pain	36	38	Stryker	Accolade	TMZF	V40	89
39	4.0	49	F	30	6	Periprosthetic fracture	28	39	Stryker	Accolade	TMZF	V40	89
40	5.2	66	F	37	N/A	N/A	28	50	Biomet	Taperloc	Ti-6Al-4V	Type I	132
41	5.0	13	F	21	3	Infection	22.2	39	Stryker	Accolade	TMZF	V40	89
42	5.2	46	F	21	7	Periprosthetic fracture	28	64	DePuy	Corail	Ti-6Al-4V	12/14	207
43	5.0	48	M	24	2	Infection	28	45	Stryker	Accolade	TMZF	V40	89
44	5.2	51	F	N/A	2	Loosening	28	34	Smith & Nephew	Spectron	CoCr	12/14	353
45	9.5	48	F	21	3	Infection	36	35	Stryker	Accolade	CoCr	V40	236
46	9.3	53	M	33	8	Component fracture	28	66	DePuy	AML	CoCr	14/16, or 12/14	374
47	10.1	51	M	32	8	Loosening	32	40	DePuy	AML	CoCr	14/16, or 12/14	676
48	13.1	64	M	29	8	PE wear	28	43	Biomet	Taperloc	Ti-6Al-4V	Type I	112
49	15.0	49	M	35	8	Periprosthetic fracture	32	52	Zimmer	Anatomic	Ti-6Al-4V	12/14	116
50	17.4	16	M	32	9	Loosening	28	36	Zimmer	Harris-Galante	Ti-6Al-4V	6°	101

BMI = body mass index; N/A = not available; F = female; M = male; PE = polyethylene; CoCr = cobalt-chromium.

Stem flexural rigidity was calculated using the equation used by Goldberg and colleagues [9]. The flexural rigidity of the stems was calculated using the Young’s modulus (E) of the alloy multiplied by the second moment of area (I = π[d]^4^/64, where d = diameter of stem at the distal contact point of the head taper). The diameters of the necks were measured by two independent observers (SBK, JAH) and were assumed to be circular. The combined lateral offset of the stem and head was obtained by tracing component markings, patient records, and component dimensional measurements or directly from the manufacturer-supplied design tables. When possible, we matched stem flexural rigidity and offset in the two cohorts using the identical stem design and size (Tables 1, 2). The stems were fabricated from a proprietary titanium alloy (54%; TMZF; Stryker Orthopaedics, Mahwah, NJ, USA) having an elastic modulus of approximately 80 GPa, Ti-6Al-4V alloy (29%; 110 GPa elastic modulus) or from a CoCr alloy (17%; 200 GPa elastic modulus).

We considered only monolithic femoral stems with a single taper interface for the head. We excluded four ceramic heads with metal sleeves from the study. Likewise, none of the CoCr heads in the matched metal head cohort included an inner modular taper adapter or sleeve.

In addition to the retrieved components, clinical data (implantation time, age, sex, body mass index [BMI], UCLA activity score, and reason for implant revision) were collected for all patients in the ceramic-metal and metal-metal taper cohorts (Tables 1 and 2, respectively). For the ceramic head cohort, the average implantation time was 3.3 ± 3.7 years (range, 0.5–18 years), the mean patient age at implantation was 52 ± 10 years, 17 of 50 (34%) were female, the mean BMI was 30 ± 7 kg/m^2^, and the mean UCLA activity score was 6 ± 2. For the metal head cohort, the average implantation time was 3.2 ± 3.8 years (range, 0.5–17 years), the mean patient age at implantation was 57 ± 14 years, 25 of 50 (50%) were female, the mean BMI was 30 ± 7 kg/m^2^, and the mean UCLA activity score was 5 ± 2 (Table 2). There was no significant difference in the implantation time (p = 0.71), sex (p = 0.11), BMI (p = 0.91), or UCLA activity levels (p = 0.65) between the ceramic and metal head cohorts. However, there was a significantly (p = 0.03) greater age in patients with a metal head as compared with the ceramic head cohort. The most frequently reported reasons for revision in both the ceramic and metal head cohorts were infection and loosening (Tables 1, 2). According to the medical records, none of the heads or stems in either the ceramic-metal or metal-metal taper cohorts was revised as a result of an adverse local tissue reaction.

### Modular Interface Damage Evaluation

Devices were cleaned in accordance with institutional procedures. The CoCr head and neck tapers were inspected visually and under a stereomicroscope equipped with a digital camera (Leica DFC490; Leica Microsystems, Wetzlar, Germany) for evidence of fretting and corrosion. Fretting, defined by Szolwinski and Farris [23] as a contact damage process resulting from micromotions of interfacing metals, was identified as scratching perpendicular to machining lines on the taper and/or wearing away of the machining lines. Corrosion was identified as white haziness (indicative of intergranular crevice corrosion), discoloration, and/or blackened debris [6].

### Scoring System for Fretting and Corrosion

Composite fretting and corrosion damage at the modular CoCr head and metal stem interfaces were characterized using a previously published 4-point scoring technique [12] with a score of 1 indicating minimal fretting or corrosion (fretting on < 10% surface and no corrosion damage); 2 indicating mild damage (fretting on > 10% surface and/or corrosion attack confined to one or more small areas); 3 indicating moderate damage (fretting > 30% and/or aggressive local corrosion attack with corrosion debris); and 4 indicating severe damage (fretting over majority [> 50%] of mating surface with severe corrosion attack and abundant corrosion debris). We analyzed metal transfer to the inner taper of the ceramic heads using a similar 4-point scoring technique with a score of 1 indicating minimal metal transfer (< 10% of the taper surface), 2 indicating metal transfer over 10%, 3 indicating metal transfer over 30%, and 4 indicating metal transfer over more than 50% of the inner head taper. We also inspected and noted the presence or absence on the inferior face of the femoral heads and the stems outside of the taper junction of dark, adherent corrosion deposits as described by Urban and colleagues [25].

The scoring plan for the head and stem tapers was developed in collaboration with a consultant biostatistician (EL). Components were randomized using a random number generator in Microsoft Excel (Microsoft Inc, Redmond, WA, USA; components were scored from the lowest to highest random number generated) and scored independently by the same three investigators (SBK, JAH, DWM). In the event of disagreement between the scores, the three investigators convened to adjudicate the discrepancy and arrive at a consensus score for the taper. The investigators were blinded to the cohort status of the stems during scoring, but it was not possible to visually score the two head cohorts in a blinded fashion.

### Scanning Electron Microscopy of Stems Interfacing With Ceramic Heads

Representative TMZF, Ti-6-4, and CoCr alloy stems, each with a visual score of 2 corresponding to the median value for the ceramic cohort, were selected for evaluation using scanning electron microscopy (SEM; JEOL 5600, Peabody, MA, USA) and energy dispersive analysis of x-rays (EDS; Princeton Gamma-Tech, Princeton, NJ, USA). Implants were either placed directly into the SEM with no additional preparation or, if too large, were sectioned distal to the taper in the neck region using a slow-speed diamond sectioning saw with water as the lubricant and then rinsed in distilled water and dried. Imaging was performed in both the backscattered and secondary electron mode and, when appropriate, EDS (Princeton Gamma-Tech) was used for elemental analysis. The primary focus of this analysis was to characterize the nature of the male taper surfaces and the type of fretting corrosion damage present.

### Statistical Analysis

Preliminary evaluation of the visual corrosion damage scoring data demonstrated a nonnormal distribution. Hence, nonparametric statistical analyses were performed using statistical software (JMP 10.0; SAS Institute, Cary, NC, USA). Mann-Whitney U, Kruskal-Wallis (with post hoc Dunn tests when necessary), and Wilcoxon tests were used to assess differences in taper damage grouped by categorical parameters (femoral head material, bearing type [for the ceramic cohort only], and ceramic material formulation [alumina versus zirconia-toughened alumina]). Spearman’s rank order correlation was used to identify correlations between continuous variables (implantation time, stem flexural rigidity, lateral offset, and head size). The level of significance chosen for all statistical analyses was p < 0.05.

## Results

**Fig. 1:**
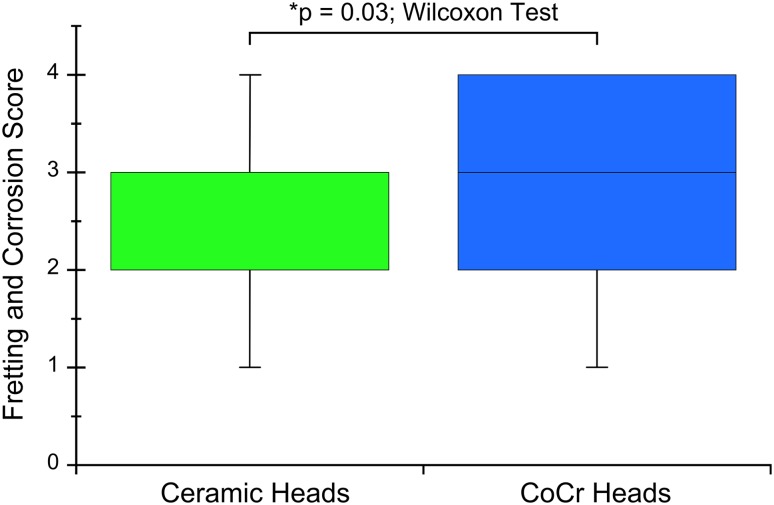
The femoral stem taper fretting and corrosion damage scores for the matched ceramic and CoCr head cohorts are shown. The damage scores were significantly lower for the ceramic cohort (p = 0.03).

**Fig. 2:**
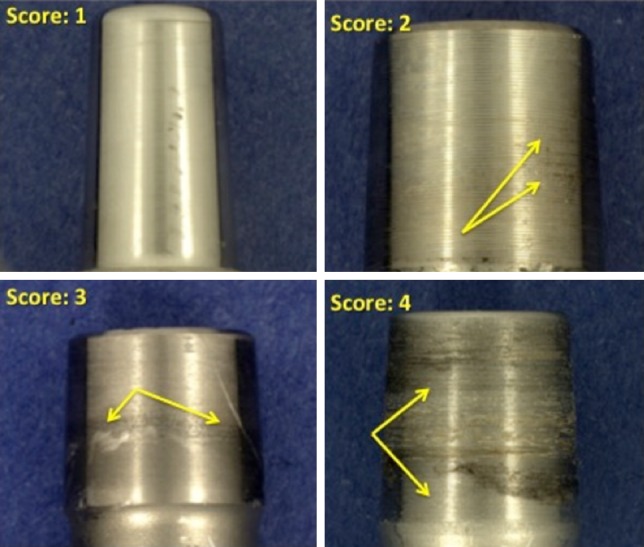
Some examples of stem taper fretting and corrosion scores for the ceramic head cohort are shown. The median score for this cohort was 2.

**Fig. 3:**
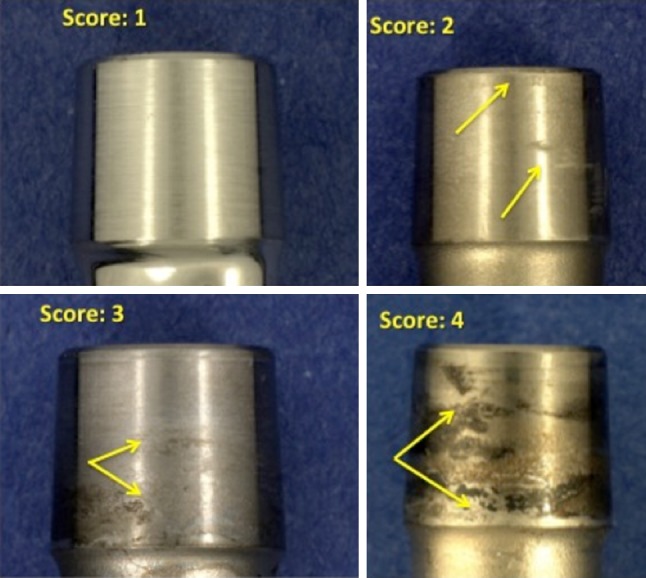
Examples of stem taper fretting and corrosion scores for the CoCr head cohort are shown. The median score for this cohort was 3.

Fretting and corrosion scores were lower for the stems in the ceramic-metal when compared with the metal-metal taper cohort (p = 0.03; Fig. 1). Evidence of fretting and corrosion, consistent with a score of 2 or greater, was observed in 42 of 50 (84%) stems in the ceramic-metal and 42 of 50 (84%) stems in the metal-metal taper cohort. The median damage score for the stems in the ceramic-metal taper cohort was 2 (Figs. 1, 2), whereas for stems in the metal-metal taper cohort, the median score was 3 (Figs. 1, 3). We observed dark corrosion deposits outside the head-neck taper junctions in three of 50 (6%) of the metal-metal taper cohort and zero of 50 (0%) of the ceramic-metal taper cohort.

**Fig. 4:**
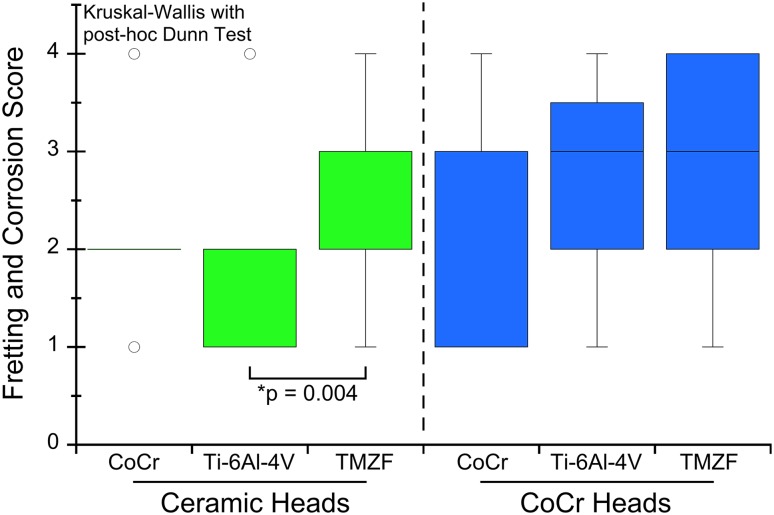
A boxplot illustrating femoral stem taper fretting and corrosion score versus stem alloy for the ceramic and metal head cohorts is presented.

Both stem alloy (p = 0.004; Kruskal-Wallis test with post hoc Dunn Test; Fig. 4) and decreased stem flexural rigidity (Spearman’s rho = −0.35, p = 0.01) were predictors of stem fretting and corrosion damage for the ceramic-metal taper cohort however, these variables did not have an effect for the metal-metal taper cohort (Fig. 4). Stem corrosion for the ceramic-metal taper cohort was not significantly affected by implantation time (p = 0.46), lateral offset (p = 0.35), head size (p = 0.26), type of ceramic bearing (p = 0.82), or the ceramic material formulation (p = 0.93). However, these tests were generally underpowered (power < 25%). The only variable in this study that was a significant predictor of the metal transfer score inside the ceramic heads was decreased flexural rigidity (Spearman’s rho = −0.35, p = 0.01). For the metal head cohort, none of the patient or device variables in this study was a significant predictor of the stem corrosion for the metal-metal taper cohort. Patient weight was positively correlated with stem fretting and corrosion scores in the ceramic head cohort (Spearman’s rho = 0.46; p = 0.002), whereas only a trend was observed in the metal head cohort (Spearman’s rho = 0.26; p = 0.08). In the metal head cohort, patient age was negatively correlated with stem fretting and corrosion scores (Spearman’s rho = −0.36; p = 0.01); however, no correlation was observed in the ceramic cohort (Spearman’s rho = 0.08; p = 0.59). Patient sex, implantation time, and activity scores were not associated with higher or lower stem fretting and corrosion scores in either cohort (p > 0.05).

**Fig. 5A–E:**
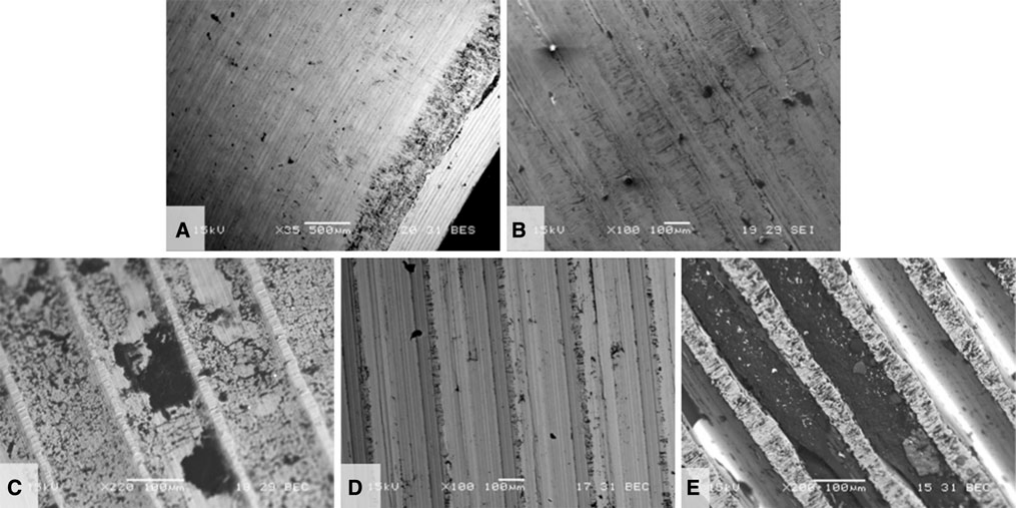
SEMs of five different design and materials for the male taper of ceramic-metal trunnions. (**A**) TMZF (Stryker Orthopaedics, Mahwah, NJ, USA) × 35 BEC, (**B**) Ti-6Al-4V (Zimmer, Inc, Warsaw, IN, USA) × 100 SEI, (**C**) Ti-6Al-4V (Wright Medical Technology, Inc, Arlington, TN, USA) × 220 BEC, (**D**) Co-Cr-Mo (DePuy Orthopaedics, Inc, Warsaw, IN, USA) × 100 BEC, (**E**) Co-Ni-Cr-Mo (Zimmer) × 100 BEC. SEI = secondary electron imaging; BEC = backscattered electron contrast image. A is a ground surface, whereas **B**–**E** have machining grooves present. Also shown are fretting scars and corrosion and biological debris present. For grooved implants, only the groove tips show evidence of fretting corrosion damage.

**Fig. 6A–D:**
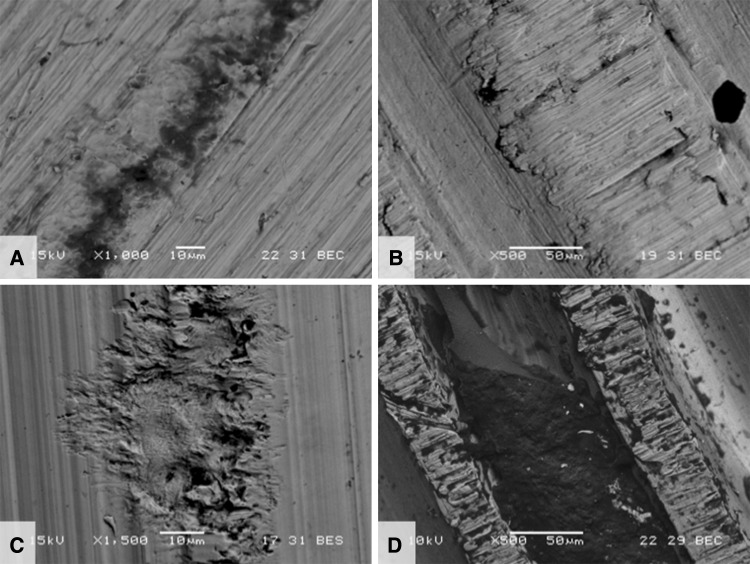
Backscattered electron micrographs of (**A**) TMZF, (**B**) Ti-6Al-4V, (**C**) Co-Cr-Mo, and (**D**) CoNiCrMo alloy tapers used in conjunction with ceramic femoral heads. Each image shows fretting damage and some corrosion debris present. In C, the damage has a distinctly corrosion-like appearance emanating from a machining ridge.

The mechanism of mechanically assisted crevice corrosion was similar in the metal and ceramic head cohorts, although in the case of ceramic femoral heads, only one of the two surfaces (the male metal taper) engaged in the oxide abrasion and repassivation process. SEM analysis showed damage on each implant that was reflective of the type of metallic surface topography present. Interestingly, the surface topography for tapers was highly variable based on alloy (Co-based or Ti-based) and manufacturer. The taper surfaces were either finely machined (TMZF; Fig. 5A) or with machined grooves present (both Ti-6Al-4V, Fig. 5B–C, and CoCr, Fig. 5D–E). The geometry of the grooves varied with design in terms of grooves per length and groove depth. For example, in Figures 5D and 5E, both implants are Co-Cr-Mo based, but Figure 5E shows tightly spaced grooves approximately 150 μm apart and roughly 60 to 100 μm deep, whereas in Figure 5D, the grooves are approximately 500 μm apart and 50 μm deep. The fretting corrosion damage seen in these tapers is intermittently distributed over the tapers and where grooves are present occur only at the top of the groove. With deep grooves, debris can accumulate (Fig. 5E) adjacent to the fretting damage. For the device in Figure 5A, the majority of fretting corrosion damage is seen in the proximal taper region (lower right of micrograph) indicating rim loading. Evidence of fretting damage and corrosion debris (dark regions) was observed on titanium alloy surfaces (Fig. 6A–B). Different types of machining grooves on cobalt alloy surfaces (Fig. 6C–D) exhibited different appearances. In one case (Fig. 6C), the damage seen has a distinct (solely) corrosion-based appearance, whereas another case (Fig. 6D) showed evidence of both fretting and corrosion damage.

## Discussion

Fretting initiated crevice corrosion observed in tapers is a complex problem and the severity is dependent on multiple factors. Retrieval studies that isolate variables in devices and patients can be designed to identify device and patient factors that aggravate or mitigate corrosion damage at the tapers. This matched cohort retrieval study was undertaken to analyze stem taper corrosion with ceramic heads as compared with CoCr heads. We theorized that ceramic femoral heads, which are electrical insulators, would lead to lower stem taper corrosion than previously reported with CoCr femoral heads; indeed, this appears to be the case. We found that decreased stem flexural rigidity and stem alloy predicted stem corrosion with modular ceramic femoral heads but not with CoCr heads. There was no difference in the mechanism of fretting corrosion between the ceramic and metal cohorts besides the fact that only the stem taper surface plays a role in the corrosion damage that occurs in the ceramic cohort.

This study had limitations. We used a matched cohort study design that was adequately powered to detect differences between the ceramic-metal and metal-metal taper cohorts, but the sample size was not sufficient to pick up correlations between taper design and secondary effects such as implantation time, which were not apparent in either cohort. The study was primarily designed to detect a difference of 1 in corrosion scores between junctions with ceramic-metal and metal-metal interfaces. However, the mean differences of fretting and corrosion scores when analyzing the device and patient factors were approximately one-fourth of what the study was designed to detect, and thus would require an unrealistically large sample size to be sufficiently powered. Because this was a secondary study question, we acknowledged that the question would be underpowered. This study also shares the same limitation of all retrieval studies, namely that they are based on analysis of clinical failures that do not necessarily reflect the population of well-functioning implants in the unrevised patient population. However, the presence of taper corrosion in this series was not associated with the reasons for revision for any of the components. This study focused on ceramic femoral heads by a single supplier with a consistent design for the past 30 years. Although we included different types of ceramic materials used in different types of bearings, we confirmed these variables did not influence the results. We also accounted for differences in stem surface finish and alloy composition between the cohorts by the matching protocol. Thus, as suggested in a study of taper corrosion with zirconia heads [10], our findings are not generalizable to other ceramic head suppliers and femoral stem designs outside of this study. Furthermore, we examined retrievals in which the only source of modularity with a metallic component was the head-stem interface. Therefore, the results of this study likewise do not apply to THA systems with multiple sources of modularity. Our results were also limited in that our methodology to assess the extent of corrosion was categorical and subjective. However, our methodology was consistent with the approach of other investigations in which corrosion and fretting of modular metallic interfaces were assessed [9]. Furthermore, it is recognized that the fretting and corrosion scoring technique does not necessarily correlate with the volume of metallic debris generated at a modular interface. Taper analyses to quantify material loss at the ceramic-stem modular connection were beyond the scope of this study.

This study demonstrates that mechanically assisted crevice corrosion can also occur in ceramic head-metal neck devices, although to a lesser extent than in CoCr head-metal neck devices. The taper designs used in these junctions were varied, but all showed evidence of some fretting and corrosion present, as expected from any modular taper connection. Despite four decades of clinical use, few studies have investigated taper corrosion involving modular ceramic heads [10, 13, 25], making comparisons with our study difficult. Urban and colleagues [25] documented one case of taper corrosion in an Autophor (Mittelmeier; Smith & Nephew, Memphis, TN, USA) hip prosthesis consisting of a CoCr femoral stem and an alumina ceramic femoral head and concluded that the corrosion products in the periprosthetic tissue and within the taper appeared to be similar to those with a CoCr head and stem. Hallab and coworkers [10] examined fretting corrosion in CoCr-CoCr and CoCr-zirconia ceramic stem-head tapers in vitro to test the hypothesis that the harder ceramic surface would result in greater fretting corrosion debris from a CoCr stem as compared with a CoCr head and stem. Contrary to their hypothesis (and similar to the results of this retrieval study), the CoCr-CoCr head-stem taper generated three- to 11-fold greater metal release than the CoCr-zirconia taper combination, but the authors cautioned against overgeneralization of their results to other head-stem designs. The manufacturer of the zirconia heads in Hallab et al.’s [10] study, St Gobain Desmarquest (Evreux Cedex, France), ultimately withdrew their product from the orthopaedic market after a worldwide recall in 2001 and they are no longer in clinical use in orthopaedics [4]. More recently, in a retrieval study of a series of titanium alloy S-ROM femoral stems (DePuy Orthopaedics, Warsaw, IN, USA), Huot Carlson et al. [13] observed less proximal femoral stem taper corrosion for cases with a ceramic-metal taper interface as opposed to cases with metal-metal taper interfaces. However, details about the design or manufacture of the ceramic heads in the S-ROM series were not reported, making direct comparisons to this study difficult [13].

The most important design and patient factors predicting increased fretting and corrosion scores of the ceramic head cohort in this study were stem material, flexural rigidity, and body weight. Previously, both in vitro and in vivo studies have found similar results [9, 13, 16, 18, 22]. We did not find lateral offset or sex to be a predictor of corrosion, which is comparable to what Hout Carlson et al. recently found [13]. Goldberg et al. [9] found that lateral offset was a predictor of corrosion; however, this factor did not have an effect when the confounding factors of flexural rigidity and implantation were considered. Head size was not a predictor for corrosion in the current study and by Hout Carlson et al. [13], which differs from a prior study that found an association between corrosion and femoral head size [11]. A post hoc power analysis revealed that this study was underpowered to detect the differences observed between head sizes (power = 21%). The clinical impact of the associated corrosion debris from these interfaces for implants with femoral heads less than 36 mm remains unclear at this point. Tissue samples were unavailable to determine the effects of these corrosion products locally and systemically.

This study provides new insight on the mechanisms of taper fretting corrosion using ceramic as an alternative to CoCr alloy femoral heads. The basic mechanism of mechanically assisted crevice corrosion was the same with the exception being that, in the case of a ceramic femoral head, only one of the two surfaces (ie, the male metal taper) engaged in the oxide abrasion and repassivation process. This, in and of itself, will lower the overall extent of corrosion. Other potential differences between taper fretting corrosion behavior could be the result of how the male taper surface was prepared. The machining topography of the metal taper appears to localize damage to the peaks of the machining grooves where contact is made with the ceramic head. However, we accounted for differences in surface topography in the two study cohorts by matching not only alloy, but stem manufacturer, where possible. Thus, the lower corrosion scores we observed between the ceramic-metal and metal-metal (not MOM, metal on metal) taper cohorts cannot be attributed to differences in surface topography. Detailed measurements of stem surface topography were also beyond the scope of the present study.

Previously, ceramic femoral heads have been discussed in the clinical literature solely in the context of an alternative bearing surface to reduce wear [1, 21]. This study has potentially important implications for modular component selection by surgeons who are concerned with Co and Cr debris release from the head-neck interface and the risk of adverse local tissue reactions [3, 8, 16–18]. Our results suggest that by using a ceramic femoral head, Co and Cr fretting and corrosion from the modular head-neck taper may be mitigated, although not completely eliminated. However, implant component selection is but one factor contributing to taper corrosion and metal debris production from modular interfaces in vivo. Taper impaction technique, engagement of the modular taper interface in a clean and dry environment, and the use of matching components are all technical factors that influence taper fretting and corrosion regardless of whether the femoral head is fabricated from CoCr or ceramic [19, 20]. Our research suggests that there could be a potentially new focus in ceramic component research in hip arthroplasty, beyond wear and tribology, to better understand the role of ceramics in mitigating modular taper corrosion.

## References

[1] Bal BS, Garino J, Ries M, Rahaman MN (2007). A review of ceramic bearing materials in total joint arthroplasty. Hip Int..

[2] Black J (1988). Does corrosion matter?. J Bone Joint Surg Br..

[3] Chana R, Esposito C, Campbell PA, Walter WK, Walter WL (2012). Mixing and matching causing taper wear: corrosion associated with pseudotumour formation. J Bone Joint Surg Br..

[4] Clarke IC, Manaka M, Green DD, Williams P, Pezzotti G, Kim YH, Ries M, Sugano N, Sedel L, Delauney C, Nissan BB, Donaldson T, Gustafson GA (2003). Current status of zirconia used in total hip implants. J Bone Joint Surg Am..

[5] Collier JP, Surprenant VA, Jensen RE, Mayor MB, Surprenant HP (1992). Corrosion between the components of modular femoral hip prostheses. J Bone Joint Surg Br..

[6] Gilbert JL, Buckley CA, Jacobs JJ (1993). In vivo corrosion of modular hip prosthesis components in mixed and similar metal combinations. The effect of crevice, stress, motion, and alloy coupling. J Biomed Mater Res..

[7] Gilbert JL, Jacobs JJ, Marlowe D, Parr J, Mayor MB (1997). The mechanical and electrochemical processes associated with taper fretting crevice corrosion: a review. Modularity of Orthopedic Implants.

[8] Gill IP, Webb J, Sloan K, Beaver RJ (2012). Corrosion at the neck-stem junction as a cause of metal ion release and pseudotumour formation. J Bone Joint Surg Br..

[9] Goldberg JR, Gilbert JL, Jacobs JJ, Bauer TW, Paprosky W, Leurgans S (2002). A multicenter retrieval study of the taper interfaces of modular hip prostheses. Clin Orthop Relat Res..

[10] Hallab NJ, Messina C, Skipor A, Jacobs JJ (2004). Differences in the fretting corrosion of metal-metal and ceramic-metal modular junctions of total hip replacements. J Orthop Res..

[11] Higgs GB, Hanzlik JA, MacDonald DW, Gilbert JL, Rimnac CM, Kurtz SM. Is increased modularity associated with increased taper and corrosion damage in metal-on-metal total hip arthroplasty devices? *J Arthroplasty.* 2013, in press. 10.1016/j.arth.2013.05.040 PMC3971476 23910820

[12] Higgs GB, Hanzlik JA, MacDonald DW, Kane WM, Day JS, Klein GR, Parviz IJ, Mont MA, Kraay MJ, Martell JM, Gilbert JL, Rimnac CM, Kurtz SM, Kurtz SM, Greenwald AS, Mihalko WM, Lemons J (2013). Method of characterizing fretting and corrosion at the various taper connections of retrieved modular components from metal-on-metal total hip arthroplasty. Metal-on-Metal Total Hip Replacement Devices, STP 1560.

[13] Huot Carlson JC, Van Citters DW, Currier JH, Bryant AM, Mayor MB, Collier JP. Femoral stem fracture and in vivo corrosion of retrieved modular femoral hips. *J Arthroplasty.* 2012;27:1389–1396.e1381. 10.1016/j.arth.2011.11.007 22209042

[14] Jacobs JJ, Gilbert JL, Urban RM (1998). Corrosion of metal orthopaedic implants. J Bone Joint Surg Am..

[15] Jacobs JJ, Urban RM, Gilbert JL, Skipor AK, Black J, Jasty M, Galante JO (1995). Local and distant products from modularity. Clin Orthop Relat Res..

[16] Langton DJ, Jameson SS, Joyce TJ, Gandhi JN, Sidaginamale R, Mereddy P, Lord J, Nargol AV (2011). Accelerating failure rate of the ASR total hip replacement. J Bone Joint Surg Br..

[17] Meneghini RM, Hallab NJ, Jacobs JJ (2012). Evaluation and treatment of painful total hip arthroplasties with modular metal taper junctions. Orthopedics..

[18] Meyer H, Mueller T, Goldau G, Chamaon K, Ruetschi M, Lohmann CH. Corrosion at the cone/taper interface leads to failure of large-diameter metal-on-metal total hip arthroplasties. *Clin Orthop Relat Res.* 2012;470:;3101–3108. 10.1007/s11999-012-2502-5 PMC3462871 22864616

[19] Mroczkowski ML, Hertzler JS, Humphrey SM, Johnson T, Blanchard CR (2006). Effect of impact assembly on the fretting corrosion of modular hip tapers. J Orthop Res..

[20] Rehmer A, Bishop NE, Morlock MM. Influence of assembly procedure and material combination on the strength of the taper connection at the head-neck junction of modular hip endoprostheses. *Clin Biomech (Bristol, Avon).* 2012;27:77–83. 10.1016/j.clinbiomech.2011.08.002 21903309

[21] Santavirta S, Bohler M, Harris WH, Konttinen YT, Lappalainen R, Muratoglu O, Rieker C, Salzer M (2003). Alternative materials to improve total hip replacement tribology. Acta Orthop Scand..

[22] Swaminathan V, Gilbert JL (2012). Fretting corrosion of CoCrMo and Ti6Al4V interfaces. Biomaterials..

[23] Szolwinski MP, Farris TN (1996). Mechanics of fretting fatigue crack formation. Wear..

[24] Urban RM, Jacobs JJ, Gilbert JL, Galante JO (1994). Migration of corrosion products from modular hip prostheses. Particle microanalysis and histopathological findings. J Bone Joint Surg Am..

[25] Urban RM, Jacobs JJ, Gilbert JL, Rice SB, Jasty M, Bragdon CR, Galante JO, Marlowe D, Parr J, Mayor MB (1997). Characterization of solid products of corrosion generated by modular-head femoral stems of different designs and materials, STP 1301. Modularity of Orthopedic Implants.

